# Potato Consumption and Risk of Cardiovascular Mortality and Type 2 Diabetes After Myocardial Infarction: A Prospective Analysis in the Alpha Omega Cohort

**DOI:** 10.3389/fnut.2021.813851

**Published:** 2022-01-27

**Authors:** Esther Cruijsen, Indira M. Indyk, Anne W. E. Simon, Maria C. Busstra, Johanna M. Geleijnse

**Affiliations:** Division of Human Nutrition and Health, Wageningen University and Research, Wageningen, Netherlands

**Keywords:** potatoes, French fries, ischemic heart disease, cardiovascular disease, mortality, type 2 diabetes mellitus, patients

## Abstract

**Background:**

Higher potato intake, especially French fries, was unfavorably associated with cardiometabolic endpoints in population-based studies. Little is known about this in patients with ischemic heart disease (IHD).

**Objective:**

Total and boiled potatoes and French fries intake were examined in relation to cardiovascular disease (CVD) mortality, all-cause mortality, and type 2 diabetes mellitus (T2DM) risk in Dutch post-myocardial infarction (MI) patients of the Alpha Omega Cohort.

**Methods:**

We analyzed 3,401 patients (60–80 years, 78% male), free from T2DM at baseline, with an MI ≤ 10 years before enrolment. Diet was assessed at baseline (2002–2006) using a 203-item validated Food Frequency Questionnaire (FFQ) that includes potato preparation methods. Cause-specific mortality was monitored through December 2018, and T2DM incidence (self-reported physician diagnosis and/or prescribed anti-diabetes medication) was monitored during the first 40 months of follow-up. Multivariable Cox models were used to obtain hazard ratios (HRs) for fatal endpoints and incident T2DM in tertiles of potato intake.

**Results:**

Patients had a median total potato intake (mainly boiled) of 111 g/d, 96% consumed >1 serving (200 g) per week. French fries were consumed by 48% of the patients (median of 6 g/d among consumers). During >12 years of follow-up (38,987 person-years), 1,476 deaths occurred of which 641 were from CVD, 394 were from IHD, and 119 were from a stroke. Total and boiled potatoes were not associated with CVD mortality, but a higher risk of all-cause mortality was observed (HR: 1.07; 95% CI: 1.01, 1.14; per 50 g/d). Potato consumption tended to be positively associated with incident T2DM (186 cases; HR: 1.11, 95% CI: 0.94, 1.32; per 50 g/d). Results for French fries were inconsistent for all outcomes.

**Conclusion:**

In Dutch post-MI patients, potatoes (mainly boiled) were not associated with CVD mortality but possibly adversely associated with all-cause mortality and T2DM risk. These findings warrant confirmation in other IHD patient cohorts. The Alpha Omega Cohort is registered at ClinicalTrials.gov as NCT03192410.

## Introduction

Potatoes are a staple food in many Western countries where they are consumed regularly in different preparations, e.g., as boiled, baked, or mashed potatoes and as French fries (1). Boiled potatoes form a major part of the traditional Dutch diet and it could be hypothesized that they have cardioprotective effects, if unsalted, because of blood pressure-lowering nutrients, such as fiber, potassium, vitamin C, and chlorogenic acid (2–5). On the other hand, potatoes have a high starch content resulting in a high glycemic index and glycemic load, which have been associated with an increased risk of type 2 diabetes mellitus (T2DM) (6, 7).

Cardiovascular disease (CVD), mainly ischemic heart disease (IHD) and stroke, and T2DM have a major impact on public health worldwide (8, 9). These cardiometabolic diseases are closely linked with T2DM being a major risk factor for CVD (8). Furthermore, in patients with CVD, T2DM is a common comorbidity and the risk of recurrent CVD is increased in patients with diabetes (10). Lifestyle habits, which include maintenance of healthy body weight, regular physical activity, and a healthy diet, form the cornerstone in the prevention and treatment of cardiometabolic diseases (8). Previous studies in patients with myocardial infarction (MI) showed that healthy eating was associated with a lower mortality and diabetes risk, on top of advanced cardiovascular drug treatment (11, 12).

Whether potatoes should be part of a healthy diet for patients with MI is not yet clear. A meta-analysis, including studies with apparently healthy populations, showed mostly neutral associations between total potato consumption and all-cause mortality, IHD and stroke risk but an increased risk of T2DM was observed (13). Especially for French fries, a 66% increased risk of T2DM was found for a daily portion of 150 g. Associations in patients with MI may be different due to medication use (e.g., statins) and alterations in the cardiovascular system. Furthermore, little is known about different preparation methods (boiled, baked, mashed, and fried) and how they would affect the health properties of potatoes. Boiling causes the leaching of nutrients and salt is often added to boiled potatoes and French fries (14, 15). Moreover, French fries contain higher amounts of fats from the frying process (16).

Therefore, we examined potato consumption with different preparation methods in relation to all-cause mortality, CVD mortality, and T2MD risk in 4,365 Dutch stable post-MI patients from the Alpha Omega Cohort.

## Methods

### Study Design

The Alpha Omega Cohort is a prospective cohort study that was originated from the Alpha Omega trial, described in detail elsewhere (17). During the initial trial phase, patients with MI were randomized to low doses of omega-3 fatty acids or placebo for a period of 40 months, which did not affect major CVD events (18). Patients have continuously been followed for cause-specific mortality since baseline (2002–2006). Written informed consent was provided by all patients, and the study was approved by a central Ethics Committee (Haga Hospital) and by the Ethics Committees of participating hospitals.

### Patients

The Alpha Omega Cohort consists of 4,837 patients, 60–80-years old at baseline with a history of MI ≤ 10 years before the start of the study. Patients with diabetes at baseline (*n* = 1,014) were excluded, as were patients with missing data on diabetes incidence (*n* = 81). Patients with missing dietary data (*n* = 325) or implausibly high or low energy intakes (<800 or >8,000 kcal/d for men, <600 or >6,000 kcal/d for women; *n* = 16) were also excluded, leaving a total of 3,401 patients for analysis (Supplementary Figure S1).

### Dietary Assessment

Baseline dietary intake of potatoes and other foods was collected using a 203-item Food Frequency Questionnaire (FFQ), which was adapted and extended from a biomarker-validated questionnaire. The Spearman correlation coefficient for reproducibility of the FFQ was 0.78 for energy intake (19). Trained dieticians checked the returned questionnaires, and additional information on unclear or missing items was obtained over the telephone. Daily intakes of foods, energy, macronutrients, and micronutrients were obtained through linkage with the Dutch Food Composition Database (NEVO) (20). Salt intake was only calculated from foods since the FFQ did not include questions on discretionary salt use during food preparation or consumption. The 2015 Dutch Healthy Diet index (DHD15-index) score was calculated to reflect adherence to dietary guidelines [DHD15-index; scale from 0 to maximal adherence (0–150)] (21).

The FFQ included questions on the frequency, amount, type of food, and preparation methods. Patients could indicate the frequency of their potato consumption separately for potato dishes, hotchpotch, and French fries in the past month ranging from “not at all” to “once per day.” The frequency of preparation methods of potatoes (boiled, mashed, and baked) and French fries (oven-fried, deep-fried, and unknown) could be indicated with “never,” “sometimes,” “often,” or “always.” Potato intake was calculated by multiplying the measured consumption frequencies with portion size (200 g for potato). The quantity of (mashed) potatoes in the composite dish “hotchpotch” was considered 48.4% based on NEVO (20). Total potato consumption (all preparation methods) was categorized into tertiles (<90.3, 90.3–129.2, >129.2 g/d). Furthermore, we categorized boiled potatoes in tertiles (<69.5, 69.5–108.4, >108.4 g/d) and analyzed French fries dichotomously (consumer vs. non-consumer).

### Other Baseline Measurements

Data on ethnicity, demographics, lifestyle, health, medical history, and anthropometrics were collected at baseline. Information was collected on the highest attained educational level (only elementary, low, intermediate, and high), smoking status (current, former, and never), and family history of diabetes (defined as having at least one parent with diabetes). Self-rated health was assessed by the question “How do you rate your overall health at this moment?” with five answer options (poor, moderate, good, very good, and excellent). Physical activity was assessed by the Physical Activity Scale for the Elderly and categorized into 3 categories: low physical activity (≤ 3 Metabolic Equivalent Tasks [METs]), intermediate physical activity [>0 to <5 days per week of moderate or vigorous activity (≥3 METs)], or high physical activity [≥5 days per week of moderate or vigorous activity (≥3 METs)] (22). Alcohol intake (g/d) was assessed by the FFQ and categorized as “no/light drinker” (men: <10 g/d, women: <5 g/d), “moderate drinker” (men: ≥10–30 g/d, women: ≥5–15 g/d), and “heavy drinker” (men: ≥30 g/d, women: ≥15 g/d). Physical examination and blood withdrawal were performed by trained research nurses at the hospital or the patient's home. Body weight and height were measured and Body Mass Index (BMI) was calculated as kg/m^2^. Obesity was defined as BMI ≥ 30. Self-reported medication use was checked by research nurses and coded according to the Anatomical Therapeutic Chemical Classification System (23). Codes were C02, C03, C07, C08, and C09 for antihypertensives, C10 for lipid-modifying drugs, and A10 for anti-diabetic drugs. Systolic blood pressure was measured two times using an automatic device, with the patient seated, after a 10-min rest (HEM-711; Omron). Hypertension was defined as systolic blood pressure ≥140 mmHg or diastolic blood pressure ≥90 mmHg. Blood lipids and glucose were analyzed by the use of standard kits and an autoanalyzer (Hitachi 912; Roche Diagnostics, Basel, Switzerland). Prevalent diabetes mellitus was defined on basis of a self-reported physician's diagnosis, use of antidiabetic medication, and/or elevated plasma glucose (≥7.0 mmol/L when fasted or ≥11.1 mmol/L when not fasted). Prediabetes was defined as plasma glucose levels ≥6.1 and <7.0 mmol/L after at least 4 h of fasting.

### Cardiometabolic Endpoints

Cardiovascular disease mortality, all-cause mortality, and T2DM incidence were the primary endpoints of this study, IHD mortality and stroke mortality were secondary endpoints. The vital status of patients was monitored through linkage with municipal registries, from baseline through December 2018. Follow-up for cause-specific mortality occurred in three phases. From 2002 to 2009 (Alpha Omega Trial), information was obtained from the national mortality registry (Statistics Netherlands, [CBS]), treating physicians, and close family members. Primary and contributing causes of death were coded by an independent Endpoint Adjudication Committee, as described previously (17, 18). After the trial from 2012, data on the primary and contributing causes of death were obtained from CBS. From 2013 onward, CBS provided data on the primary cause of death only, and treating physicians were asked to fill out an additional cause-of-death questionnaire (response rate: 67%), which was coded by study physicians who were not involved in the current analysis. The endpoint CVD, IHD, or stroke was allocated to all patients for whom it was a primary or contributing cause of death, based on any of the data sources. Mortality coding was performed according to the International Classification of Diseases, tenth revision (ICD-10) (24). CVD mortality comprised ICD-10 codes I20–I25 (IHD), I46 (cardiac arrest), R96 (sudden death, undefined), I50 (heart failure), and I60–I69 (stroke). IHD mortality comprised I20–I25, I46, and R96. Stroke mortality comprised I60–I69.

Type 2 diabetes mellitus was ascertained based on a self-reported physician's diagnosis and/or the initiation of antidiabetic medication after 12 months, at midterm examination (20 months), 24 months, and at the end of the Alpha Omega Trial phase (40 months) through questionnaires and telephone calls. Patients reported the date of diagnosis or start of medication use. If this information was missing, the midpoint between two interview dates was used as the date of T2DM incidence. T2DM incidence was not based on plasma glucose concentrations because blood samples were only collected for part of the cohort during follow-up.

### Statistical Analysis

Baseline patient characteristics are presented across tertiles of total potato consumption as mean ± SD for normally distributed variables, median (interquartile range; IQR) for variables with a skewed distribution, and as *n* (%) for categorical variables. Missing data (assuming missing at random) were imputed using the age- and sex-specific mode for physical activity (*n* = 25), smoking status (*n* = 1), family history of diabetes (*n* = 65), education level (*n* = 15), and self-rated health (*n* = 11) and the age- and sex-specific median for BMI (*n* = 6) to retain these patients in the multivariable models. Total potato and boiled potato intake (except for French Fries which were dichotomous) was adjusted for total energy using the residual method by Willett et al. (25). Associations of total potato consumption, boiled potatoes, and French fries with CVD, all-cause, IHD, and stroke mortality and incident T2DM were assessed using Cox proportional hazards models. Hazard ratios (HRs) with 95% CIs are presented in tertiles of total and boiled potato intake (using the lowest tertile as the reference) and continuously, per 50 g/d. For French fries, HRs (95% CIs) are presented in consumers vs. non-consumers. Log-minus-log plots were used to visually check the proportionality of hazards assumption which was met. For mortality outcomes, survival time was calculated from the date of study enrollment to death or end of the study (December 31, 2018). One patient was lost to follow-up and censored after 2.9 years. For incident T2DM, survival time was calculated from the date of study enrollment to the date of T2DM diagnosis, death, or end of the 40th follow-up period.

Hazard ratios in the first model were adjusted for age (y), sex, and total energy intake (kJ). The second model was additionally adjusted for education level (4 categories), smoking (3 categories), physical activity (3 categories), alcohol (3 categories), and family history of diabetes (yes/no family history; only for T2DM incidence). The third model was additionally adjusted for whole and refined grains, fish, red and processed meat, milk, yogurt and custard, vegetables, fruits, mayonnaise, saturated fatty acids, polyunsaturated fatty acids, sugar-sweetened beverages, sweet and savory snacks and nuts, and seeds and legumes. When analyzing boiled potatoes, the third model was additionally adjusted for non-boiled potato dishes and when analyzing French fries, the model was additionally adjusted for non-fried potato dishes. HRs were in an extra analysis additionally adjusted for the DHD15-index. Restricted cubic splines (RCSs) were used to investigate the continuous association between total potatoes with CVD mortality, all-cause mortality, and T2DM, using the fully adjusted model. The median intake was set as the reference, and knots were placed at the 5th, 50th, and 95th percentiles. The non-linearity of associations was assessed using the Wald chi-square test (26).

Subgroup analyses were performed for CVD mortality, all-cause mortality, and T2DM, for sex (men/women), prevalent obesity (obese/non-obese), smoking (smoker/non-smoker), fiber intake (<20/≥ 20 g/d), physical activity (low/moderate and high), self-rated health (poor and moderate/good and excellent), and diet quality (low/high based on median DHD15-index). For T2DM, an additional subgroup analysis was performed for family history of diabetes mellitus (yes/no). For CVD and all-cause mortality, sensitivity analyses were performed by excluding the first 2 years of follow-up and excluding 479 patients with MI <1-year prior enrollment. We also performed sensitivity analyses with shortened follow-up duration from December 31, 2012 (median: 7.4 years). For T2DM, a sensitivity analysis was performed excluding 405 patients with prediabetes. Two-sided values of *p* < 0.05 were considered statistically significant. SAS statistical software was used to perform all analyses (version 9.4; SAS Institute Inc, Cary, NC, USA). Forest plots were created using R version 3.6.1 (R Foundation for Statistical Computing).

## Results

Table 1 presents baseline characteristics for the whole cohort and across tertiles of energy-adjusted total potato consumption. The mean age of patients was 68.9 ± 5.5 years, 20% were male and most patients were Dutch. The average BMI was 27.4 ± 3.5 and 20% were obese. The majority of patients used antihypertensive medication (89%) and/or statins (87%). Most patients (99%) consumed potatoes, 96% consumed potatoes at least one time per week. Median daily intakes were 111 g (3.8 weekly servings of 200 g) for total potatoes and 85 g for boiled potatoes. Median French fries intake in consumers (*n* = 1,622; 48%) was 6.2 g/d, which corresponds to approximately 1 serving a month. During long-term follow-up (median: 12.5 years; 38,987 person-years) 1,476 deaths occurred, i.e., 641 from CVD, 394 from IHD, and 119 from a stroke. During the early follow-up phase of 40 months, 186 incident T2DM cases have occurred.

**Table 1:** Baseline characteristics of 3,401 patients of the Alpha Omega Cohort, overall, and in tertiles of total potato intake^a^.

	**Total** **(*n* = 3,401)**	**Energy-adjusted total potato intake**		
		**Tertile 1**	**Tertile 2**	**Tertile 3**
		**(*n* = 1,133) <90.3 g/d**	**(*n* = 1,134) 90.3–129.2 g/d**	**(*n* = 1,134) >129.2 g/d**
Age, year	68.9 ± 5.5	68.5 ± 5.6	68.8 ± 5.4	69.3 ± 5.5
Females	680 (20)	277 (24)	204 (18)	199 (18)
Dutch ethnicity	3,359 (99)	1,114 (98)	1,126 (99)	1,128 (99)
BMI^b^, kg/m^2^	27.4 ± 3.5	27.3 ± 3.6	27.3 ± 3.4	27.4 ± 3.4
Obese	685 (20)	239 (21)	217 (19)	229 (20)
Body weight^c^, kg	81.6 ± 12.0	80.7 ± 12.3	81.9 ± 11.7	82.0 ± 11.8
Educational level^d^				
Only elementary	667 (20)	175 (15)	227 (20)	265 (23)
Low	1,221 (36)	381 (34)	401 (36)	439 (39)
Intermediate	1,070 (32)	396 (35)	367 (33)	307 (27)
High	428 (13)	177 (16)	134 (12)	117 (10)
Smoking status^e^				
Never	572 (17)	200 (18)	176 (16)	196 (17)
Former	2278 (67)	736 (65)	783 (69)	759 (67)
Current	550 (16)	196 (17)	175 (15)	179 (16)
Physical activity^f^				
Low	1,314 (39)	427 (38)	424 (37)	463 (41)
Intermediate	723 (21)	271 (24)	222 (20)	230 (20)
High	1,347 (40)	430 (38)	484 (43)	433 (38)
Self-rated health^g^				
Very good or excellent	440 (13)	147 (13)	155 (14)	138 (12)
Good	2,254 (66)	714 (63)	765 (68)	775 (69)
Moderate or poor	696 (21)	266 (23)	212 (19)	218 (19)
Alcohol intake				
No or light drinker	1,819 (53)	583 (51)	587 (52)	649 (57)
Moderate drinker	1,011 (30)	329 (29)	349 (31)	333 (29)
Heavy drinker	571 (17)	221 (20)	198 (17)	152 (13)
Time since last myocardial infarction, y^h^	3.5 (1.6–6.3)	3.2 (1.5–5.3)	3.5 (1.6–6.1)	4.0 (1.6–6.6)
Family history of diabetes	559 (16)	178 (16)	178 (16)	203 (18)
Prevalent prediabetes^i^	210 (7)	75 (16)	65 (6)	70 (7)
Plasma glucose, ^j^ mmol/L	5.6 ± 1.0	5.6. ± 0.9	5.6 ± 1.0	5.6 ± 1.0
Blood pressure,^k^ mmHg				
Systolic	141.7 ± 21.5	141.6 ± 21.8	141.1 ± 21.1	142.3 ± 21.6
Diastolic	80.7 ± 11.1	81.2 ± 11.0	80.4 ± 11.2	80.7 ± 11.2
Hypertension^l^	3,228 (95)	1,074 (95)	1,077 (95)	1,077 (95)
Serum lipids, mmol/L				
LDL cholesterol^m^	2.6 ± 0.8	2.6 ± 0.8	2.6 ± 0.8	2.6 ± 0.8
HDL cholesterol^n^	1.3 ± 0.3	1.3 ± 0.3	1.3 ± 0.3	1.3 ± 0.3
Use of cardiovascular medication				
Antihypertensive medication	3,024 (89)	1,009 (89)	1,012 (89)	1,003 (88)
Lipid-modifying medication	2,953 (87)	974 (86)	1,007 (89)	972 (86)
Total energy intake, kJ/d	8,170 ± 2,210	8,158 ± 2,451	8,301 ± 2,043	8,052 ± 2,112
Intake dietary factors^o^, g/d				
Boiled potato	74 (46–99)	37 (25–50)	74 (60–99)	105 (84–140)
Baked potato	0 (0–24)	0 (0–12)	12 (0–24)	0 (0–33)
Mashed potato	0 (0–0)	0 (0–3)	0 (0–0)	0 (0–0)
Hotchpot	20 (0–59)	17 (0–31)	20 (0–39)	39 (17–61)
French fries	0 (0–6)	0 (0–4)	2 (0–8)	0 (0–8)
Whole grains	118 (88–158)	118 (88–158)	125 (88–161)	121 (88–157)
Refined grains	41 (21–71)	42 (21–74)	40 (21–72)	41 (20–70)
Fish	14 (5–18)	15 (6–23)	15 (5–15)	12 (4–17)
Red and processed meat	69 (43–94)	63 (40–91)	70 (45–95)	72 (43–97)
Milk	150 (58–103)	150 (21–150)	150 (21–150)	150 (21–150)
Yogurt and custard	107 (21–150)	107 (21–150)	107 (21–150)	107 (21–150)
Vegetables	78 (58–103)	62 (43–91)	78 (63–97)	92 (73–117)
Fruits	110 (43–249)	114 (44–259)	109 (43–253)	106 (43–219)
Nuts, seeds and legumes	8 (4–14)	9 (4–14)	8 (4–14)	8 (4–14)
Cooking fats	10 (2–23)	7 (1–20)	12 (3–25)	11 (2–25)
Mayonnaise	0 (0–1)	0 (0–1)	0 (0–1)	0 (0–1)
Sugar-sweetened beverages	127 (46–210)	146 (54–237)	127 (42–203)	107 (41–188)
Sweet snacks	55 (31–86)	58 (32–93)	58 (35–86)	50 (29–76)
Savory snacks	16 (9–28)	17 (9–29)	17 (9–29)	16 (8–27)
Saturated fatty acids	27 (21–34)	27 (20–35)	27 (22-35)	26 (20–30)
Poly unsaturated fatty acids	14 (11-20)	13 (10-19)	15 (11-21)	15 (11-20)
Sodium^p^	2.1 (1.7–2.6)	2.1 (1.7–2.7)	2.2 (1.8–2.7)	2.1 (1.7–2.6)
DHD15-index score	79 ± 14	78 ± 14	79 ± 13	81 ± 13

a *Values are means ± SDs for normally distributed variables, medians (IQRs) for skewed variables, or n (%) for categorical variables unless otherwise indicated. DHD15-index, Dutch Healthy Diet 2015 index; MET, metabolic equivalent task.*

b *Missing data for 4 patients. Obesity was defined as BMI ≥ 30 kg/m^2^.*

c *Missing data for 2 patients.*

d *Missing data for 15 patients.*

e *Missing data for 1 patient.*

f *Missing data for 27 patients. Low activity is defined as ≤ 3 METs, intermediate activity as >3 METs on > 0 to <5 days per week, and high activity as >3 METs on ≥ days per week.*

g *Missing data for 6 patients.*

h *Missing data for 23 patients. Myocardial infarction was based on a verified clinical diagnosis <10 years before study enrolment.*

i *Missing data for 195 patients. Defined as plasma glucose levels ≥ 6.1 and <7.0 mmol/L after at least 4 h of fasting.*

j *Non-fasted, missing data for 60 patients.*

k *Missing data for 4 patients.*

l *Defined as systolic blood pressure ≥ 140 mmHg or diastolic blood pressure ≥ 90 mmHg.*

m *Non-fasted, missing data for 198 patients.*

n *Non-fasted, missing data for 76 patients.*

o *Unadjusted for total energy intake.*

p *Sodium intake was only estimated from foods, since discretionary salt use could not be assessed by means of the FFQ*.

### CVD Mortality

**Figure 1:**
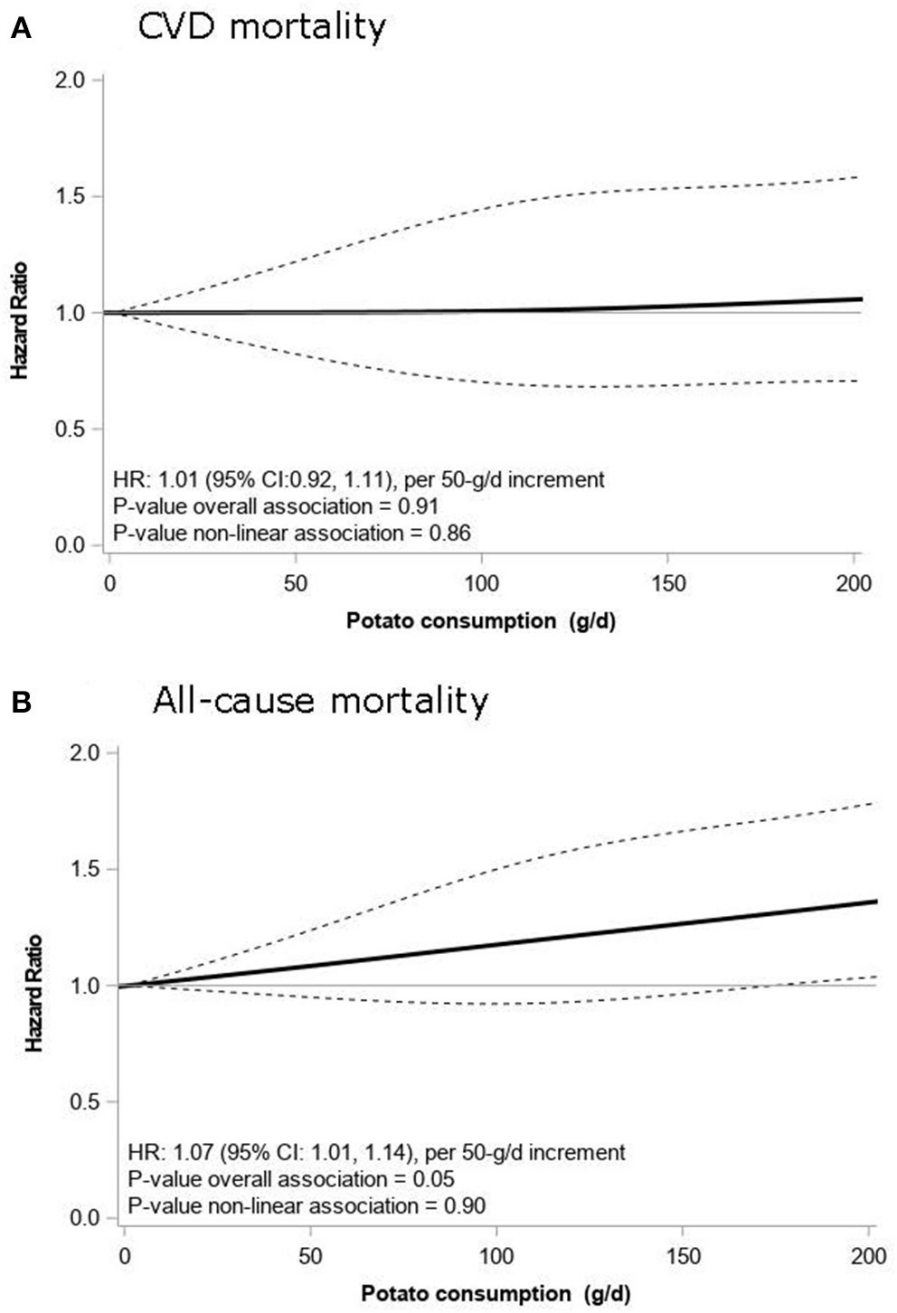
Associations of total potato intake with CVD mortality **(A)** and all-cause mortality **(B)** in 3,401 patients from the Alpha Omega Cohort. Lines are restricted cubic splines, showing continuous associations, with 3 knots located at the 5th, 50th, and 95th percentiles. The y-axis shows the predicted hazard ratios (HRs) for mortality for any value of total potato intake, compared to the reference value set at 0 g/d. HRs are adjusted for age, sex, energy intake, education level, smoking, physical activity level, alcohol intake, whole and refined grains, fish, red and processed meat, milk, yogurt and custard, vegetables, fruits, mayonnaise, saturated fatty acids, polyunsaturated fatty acids, sugar-sweetened beverages, sweet and sour snacks and nuts, seeds, and legumes. CVD, cardiovascular disease; HR, hazard ratio.

Table 2 presents HRs for total potato consumption in relation to CVD, IHD, and stroke mortality. Total potato consumption showed no association with CVD mortality with HRs (95% CIs) of 1.00 (0.82, 1.22) for Tertile 2 and 1.00 (0.81, 1.24) for Tertile 3 compared to Tertile 1. When analyzed continuously, total potato intake (per 50 g) was also not associated with CVD mortality (HR: 1.01; 95% CI: 0.92, 1.11; Figure 1). Similarly, no associations were observed for CVD mortality with boiled potatoes (HR: 1.03; 95% CI: 0.93, 1.13; per 50 g increment; Table 3) and French fries (HR: 0.89; 95% CI: 0.74, 1.06; consumer vs. non-consumer; Table 3). Results for IHD mortality were in line with CVD mortality showing no associations for total potato intake (HR: 0.96; 95% CI: 0.85, 1.08; per 50 g increment), boiled potatoes (0.99; 95% CI: 0.88, 1.12; per 50 g increment), and French fries (HR: 0.93; 95% CI: 0.74, 1.16; consumer vs. non-consumer). Similarly for stroke mortality, no associations were observed for total potato intake (HR: 1.00; 95% CI: 0.81, 1.24; per 50 g increment), boiled potatoes (HR: 1.00; 95% CI: 0.80, 1.25; per 50 g increment), and French fries (HR: 0.94; 95% CI: 0.62, 1.41; consumer vs. non-consumer). Additional adjustment for the DHD15-index did not change the HRs (data not shown).

**Table 2:** Hazard ratios (HRs) for total potato intake in relation to CVD, all-cause, IHD, stroke mortality, and T2DM incidence in 3,401 patients from the Alpha Omega Cohort^a^.

	**Energy-adjusted total potato intake**			
	**Tertile 1**	**Tertile 2**	**Tertile 3**	**Per 50 g increment**
	**(*n* = 1,133)**	**(*n* = 1,134)**	**(*n* = 1,134)**	
	** <90.3 g/d**	**90.3–129.2 g/d**	**>129.2 g/d**	
Median (IQR) potato intake, g/d	66 (50–78)	111 (101–120)	153 (140–174)	111 (78–140)
**CVD mortality**				
Person-years	13,002	13,260	12,724	38,987
Cases	212	213	216	641
Model 1^b^, ^c^	1.00	0.94 (0.77, 1.13)	0.95 (0.79, 1.15)	0.98 (0.91, 1.07)
Model 2^c^, ^d^	1.00	0.96 (0.79, 1.16)	0.95 (0.78, 1.15)	0.98 (0.90, 1.06)
Model 3^c^, ^e^	1.00	1.00 (0.82, 1.22)	1.00 (0.81, 1.24)	1.01 (0.92, 1.11)
**All-cause mortality**				
Cases	470	468	538	1,476
Model 1^b^, ^c^	1.00	0.93 (0.81, 1.05)	1.07 (0.94, 1.21)	1.06 (1.00, 1.11)
Model 2^c^, ^d^	1.00	0.94 (0.83, 1.07)	1.06 (0.94, 1.21)	1.05 (1.00, 1.11)
Model 3^c^, ^e^	1.00	0.97 (0.85, 1.10)	1.09 (0.95, 1.25)	1.07 (1.01, 1.14)
**IHD mortality**				
Cases	134	135	125	394
Model 1^b^, ^c^	1.00	0.94 (0.74, 1.20)	0.87 (0.68, 1.12)	0.94 (0.84, 1.04)
Model 2^c^, ^d^	1.00	0.97 (0.77, 1.24)	0.87 (0.68, 1.11)	0.94 (0.84, 1.04)
Model 3^c^, ^e^	1.00	1.02 (0.79, 1.30)	0.91 (0.69, 1.19)	0.96 (0.85, 1.08)
**Stroke mortality**				
Cases	38	41	40	119
Model 1^b^, ^c^	1.00	1.01 (0.65, 1.58)	1.00 (0.64, 1.56)	0.95 (0.78, 1.15)
Model 2^c^, ^d^	1.00	1.05 (0.68, 1.64)	1.02 (1.65, 1.60)	0.95 (0.79, 1.15)
Model 3^c^, ^e^	1.00	1.15 (0.73, 1.83)	1.16 (0.71, 1.90)	1.00 (0.81, 1.24)
**T2DM**				
Person-years	3,628	3,635	3,597	10,860
Cases	50	65	71	186
Model 1^b^, ^c^	1.00	1.29 (0.89, 1.87)	1.43 (0.99, 2.06)	1.07 (0.93, 1.24)
Model 2^c^, ^f^	1.00	1.32 (0.91, 1.92)	1.45 (1.00, 2.09)	1.07 (0.93, 1.24)
Model 3^c^, ^e^	1.00	1.37 (0.93, 2.00)	1.54 (1.04, 2.30)	1.10 (0.93, 1.30)

a *CVD, cardiovascular disease; IHD, ischemic heart disease; T2DM, type 2 diabetes mellitus.*

b *Adjusted for age, sex, and total energy intake.*

c *Values are HRs (95% CIs) obtained from Cox proportional hazards models, using Tertile 1 as reference.*

d *Adjusted as in Model 1, plus for education level, smoking, physical activity, and alcohol intake.*

e *Adjusted as in Model 2, plus for whole and refined grains, fish, red and processed meat, milk, and yogurt and custard, vegetables, fruits mayonnaise, saturated fatty acids, polyunsaturated fatty acids, sugar-sweetened beverages, sweet and sour snacks and nuts, seeds, and legumes.*

f *Adjusted as in Model 1, plus for education level, smoking, physical activity, alcohol intake, and family history of diabetes mellitus*.

**Table 3:** Hazard ratios for boiled potato intake and French fries in relation to CVD, all-cause, IHD, stroke mortality, and T2DM incidence in 3,401 patients from the Alpha Omega Cohort^a^.

	**Energy-adjusted boiled potato intake**				**French fries**	
	**Tertile 1**	**Tertile 2**	**Tertile 3**	**Per 50 g increment**	**Non-consumer**	**Consumer (*n* = 1,622)**
	**(*n* = 1,133)**	**(*n* = 1,134)**	**(*n* = 1,134)**		**(*n* = 1,779)**	
	**<90.3 g/d**	**90.3–129.2 g/d**	**>129.2 g/d**			
Median (IQR) potato intake, g/d	45 (29–57)	90 (80–99)	133 (119–153)	90 (58–119)	0	6.2 (3.5–13.3)
**CVD mortality**						
Person-years	13,002	13,260	12,724	38,987	19,882	19,104
Cases	212	213	216	641	377	264
Model 1^b^, ^c^	1.00	0.96 (0.79, 1.16)	0.99 (0.81, 1.20)	1.00 (0.92, 1.09)	1.00	0.88 (0.75, 1.04)
Model 2^c^, ^d^	1.00	0.97 (0.80, 1.18)	0.97 (0.80, 1.19)	1.00 (0.91, 1.08)	1.00	0.90 (0.76, 1.05)
Model 3^c^, ^e^	1.00	1.02 (0.93, 1.24)	1.03 (0.83, 1.28)	1.03 (0.93, 1.13)	1.00	0.89 (0.74, 1.06)
**All-cause mortality**						
Cases	470	468	538	1,476	868	608
Model 1^b^, ^c^	1.00	0.95 (0.83, 1.08)	1.11 (0.97, 1.26)	1.08 (1.02, 1.14)	1.00	0.88 (0.79, 0.98)
Model 2^c^, ^d^	1.00	0.96 (0.84, 1.09)	1.10 (0.96, 1.25)	1.07 (1.01, 1.13)	1.00	0.89 (0.80, 0.99)
Model 3^c^, ^e^	1.00	0.96 (0.84, 1.09)	1.10 (0.96, 1.25)	1.09 (1.03, 1.16)	1.00	0.88 (0.79, 1.00)
**IHD mortality**						
Cases	134	135	125	394	230	164
Model 1^b^, ^c^	1.00	0.99 (0.78, 1.26)	0.94 (0.73, 1.20)	0.97 (0.87, 1.08)	1.00	0.90 (0.73, 1.11)
Model 2^c^, ^d^	1.00	1.01 (0.79, 1.29)	0.93 (0.72, 1.20)	0.97 (0.87, 1.08)	1.00	0.91 (0.74, 1.12)
Model 3^c^, ^e^	1.00	1.05 (0.82, 1.35)	0.96 (0.73, 1.27)	0.99 (0.88, 1.12)	1.00	0.93 (0.74, 1.16)
**Stroke mortality**						
Cases	38	41	40	119	66	53
Model 1^b^, ^c^	1.00	1.01 (0.64, 1.58)	0.99 (0.63, 1.57)	0.94 (0.77, 1.15)	1.00	0.98 (0.67, 1.42)
Model 2^c^, ^d^	1.00	1.05 (0.67, 1.64)	1.01 (0.63, 1.60)	0.94 (0.77, 1.15)	1.00	1.01 (0.69, 1.46)
Model 3^c^, ^e^	1.00	1.16 (0.73, 1.84)	1.16 (0.70, 1.93)	1.00 (0.80, 1.25)	1.00	0.94 (0.62, 1.41)
**T2DM**						
Person-years	3,628	3,635	3,597	10,860	5,708	5,153
Cases	50	65	71	186	85	101
Model 1^b^, ^c^	1.00	1.30 (0.90, 1.89)	1.45 (1.00, 2.10)	1.07 (0.92, 1.24)	1.00	1.34 (1.00, 1.81)
Model 2^c^, ^f^	1.00	1.33 (0.92, 1.94)	1.47 (1.01, 2.14)	1.08 (0.92, 1.25)	1.00	1.34 (0.99, 1.80)
Model 3^c^, ^d^	1.00	1.39 (0.95, 2.05)	1.60 (1.06, 2.40)	1.11 (0.94, 1.32)	1.00	1.22 (0.87, 1.69)

a *CVD, cardiovascular disease; IHD, ischemic heart disease; T2DM, type 2 diabetes mellitus.*

b *Adjusted for age, sex, and total energy intake.*

c *Values are HRs (95% CIs) obtained from Cox proportional hazards models, using Tertile 1 as reference.*

d *Adjusted as in Model 1, plus for education level, smoking, physical activity, and alcohol intake.*

e *Adjusted as in Model 2, plus for whole and refined grains, fish, red and processed meat, milk, yogurt and custard, vegetables, fruits, mayonnaise, saturated fatty acids, polyunsaturated fatty acids, sugar-sweetened beverages, sweet and sour snacks and nuts, seeds, and legumes. For boiled potatoes, additionally adjusted for non-boiled potato dishes. For French fries, additionally adjusted for non-fried potato dishes.*

f *Adjusted as in Model 1, plus for education level, smoking, physical activity, alcohol intake, and family history of diabetes mellitus*.

### All-Cause Mortality

Table 2 presents HRs for total potato consumption in relation to all-cause mortality. HRs were 1.02 (95% CI: 0.79, 1.30) for Tertile 2 and 1.09 (95% CI: 0.95, 1.25) for Tertile 3 compared to the lowest tertile, using the fully adjusted model. When analyzed continuously, a linear association was observed with a 7% higher risk of all-cause mortality per 50-g/d increment in total potato intake (HR: 1.07; 95% CI: 1.01, 1.14; Table 2, Figure 1). Boiled potato intake was also associated with a higher risk of all-cause mortality with an HR of 1.09 (95% CI: 1.03, 1.16) per 50-g/d increment (Table 3). French fries intake was borderline significantly associated with a lower risk of all-cause mortality (HR: 0.88; 95% CI: 0.79, 1.00; consumer vs. non-consumer).

### Type 2 Diabetes Incidence

Table 2 presents HRs for total potato consumption in relation to T2DM incidence. Potato intake in the highest tertile was associated with a higher risk of T2DM (HR: 1.54; 95% CI: 1.04, 2.30) compared to the lowest tertile. When analyzed continuously, the HR per 50-g/d increment of total potato intake was 1.10 (95% CI: 0.93, 1.30; Supplementary Figure S2). The highest tertile of boiled potato consumption was also associated with a higher T2DM risk (HR: 1.60; 95% CI: 1.06, 2.40) as compared to Tertile 1. The HR per 50-g/d of boiled potato intake was 1.11 (95% CI: 0.94, 1.32). The HR for French fries was 1.22 (95% CI: 0.87, 1.69) when comparing consumers with non-consumers (Table 3).

### Subgroup and Sensitivity Analyses

Supplementary Figures S3–S5 present associations of total potato consumption per 50-g/d increment with CVD mortality, all-cause mortality, and T2DM across several subgroups. Results for CVD mortality with total potato intake were not essentially modified by sex, obesity status, smoking status, fiber intake, physical activity level, self-rated health, and diet quality with large overlapping confidence intervals between subgroups (Supplementary Figure S3).

For all-cause mortality, no association was shown with potato intake in patients with obesity (HR: 0.98; 95% CI: 0.86, 1.12) while the association remained in patients without obesity (HR: 1.10; 95% CI: 1.03, 1.17; Supplementary Figure S4). Moreover, no association was observed for patients with poor/moderate self-rated health (HR: 0.98; 95% CI: 0.87, 1.10) while the association remained in patients with good/excellent self-rated health (HR: 1.11; 95% CI: 1.04, 1.19). Results for all-cause mortality were not essentially modified by sex, smoking status, fiber intake, physical activity level, and diet quality.

For T2DM, associations were more pronounced in patients with obesity (HR: 1.28; 95% CI: 0.79, 2.06), a high fiber intake (HR: 1.29; 95% CI: 0.96, 0.74), poor/moderate self-rated health (HR: 1.35; 95% CI: 0.95, 1.91), and a family history of diabetes (HR: 1.29; 95% CI: 0.86, 1.93), but findings were not statistically significant (Supplementary Figure S5). Results for T2DM were not essentially modified by sex, smoking status, physical activity level, and diet quality.

Excluding the first 2 years of follow-up and excluding patients with an MI <1-year prior enrollment and using a shortened follow-up duration did not change the results for CVD mortality and all-cause mortality (Supplementary Figures S3, S4). Excluding patients with prediabetes did not change the results for T2DM (Supplementary Figure S5).

## Discussion

In this prospective analysis of 3,401 Dutch post-MI patients with a relatively high intake of potatoes (mainly boiled), no associations were found between potato consumption and CVD mortality during >10 years of follow-up. For all-cause mortality, however, a possible ≈25% higher risk was observed in patients with a potato intake of 150 g/d or more, compared to non-consumers. Potato consumption tended to be positively associated with incident T2DM during 40 months of follow-up. The intake of French fries, for which consumption levels were low, was inconsistently related to the different outcomes.

Data on potato consumption and CVD mortality in patients with MI are lacking, but several observational studies have been conducted in the general population. In a Swedish population-based cohort (*n* = 69,313) with 13 years of follow-up, the relative risk of CVD mortality for 3 servings per week of total potato consumption (boiled/fried potatoes and French fries) was 1.00 (95% CI: 0.97, 1.02) (27), which is in line with our results. In the Greek subcohort of the European Prospective Investigation into Cancer and Nutrition (EPIC) study in 23,929 healthy adults with a median potato intake around 80 g/d, non-significant HRs of 0.92 in men and 1.15 in women were found, for 10-year risk of IHD mortality also no relationship was found (28). In the Alpha-Tocopherol, Beta-Carotene Cancer Prevention (ATBC) cohort of 21,930 Finnish male smokers, an inverse association of potato consumption with IHD mortality was found, with a relative risk of 0.74 (95% CI: 0.57, 0.97) for intakes above ≈250 g/d. However, apart from energy and alcohol intake, no adjustment for dietary confounders was made in that study (29). In the Nurses' Health Study (*n* = 66,719) and Health Professionals' Follow-up Study (*n* = 42,016), potato consumption was not associated with CVD mortality (all HRs ≈ 1.00) (30). Overall, currently available evidence suggests that potato consumption does not play a major role in the development of (recurrent) CVD.

The intake of total and boiled potatoes was possibly associated with a higher risk of all-cause mortality (7–9% per 50-g/d, respectively) in our cohort of Dutch post-MI patients. Prospective studies on potato intake and overall mortality are lacking in patients with MI, but several studies have been conducted in the general population. A meta-analysis by Schwingshackl et al. of population-based cohorts from the USA and Europe (26,775 participants) yielded a pooled Relative Risk (RR) of 0.97 (95% CI: 0.86, 1.10) when comparing extreme potato intake categories, or 0.88 (95% CI: 0.69, 1.12) per 150 g/d (13). A recent analysis in the Nurses' Health Study and Health Professionals' Follow-up Study, not included in the meta-analysis by Schwingshackl, showed no associations with all-cause mortality risk (all HRs ≈ 1.0) (30). We have no plausible explanation for the higher risk of all-cause mortality in relation to (boiled) potatoes in the Alpha Omega Cohort. As discussed above, the risk of CVD mortality, comprising 43% of all fatal cases, was not related to potato intake. Around 30% of deaths in our cohort were due to cancers, such as lung cancer and colorectal cancer. A meta-analysis of four population-based cohorts showed no association between potato consumption and cancer mortality (RR: 1.05; 95% CI: 0.99, 1.12, per 100-g/d) (31). Studies of potato intake and cancer mortality in patients with IHD, however, are lacking for comparison to our data. We found an unexpected inverse association for French fries with all-cause mortality, in contrast to findings from population-based studies. In >4,000 healthy US adults, for example, RRs for all-cause mortality increased from 1.37 (95% CI: 0.99, 1.91) for 2–3 monthly servings of French fries to 2.26 (95% CI: 1.15, 4.47) for ≥3 weekly servings, compared to zero intake (32). In our study, French fries were consumed by only half of the cohort, and intake among consumers was low (≈1 serving a month), possibly due to under-reporting.

Potatoes are naturally high in starches (60–80% in dry matter) and a source of dietary fiber and micronutrients (e.g., potassium, vitamin B6, and vitamin C) (33). A high intake of starch from potatoes has been linked to hyperglycemia and T2DM risk (34). Furthermore, potato consumption has been linked to weight gain, the main risk factor for T2DM (34). Schwingshackl et al. examined potato intake and T2DM risk in a meta-analysis of seven population-based cohorts from the USA, Europe, Australia, and China, with a total of 18,334 incident T2DM cases (13). RRs (per 150 g/d) were 1.18 (95% CI: 1.10, 1.27) for total potatoes, 1.09 (95% CI: 1.01, 1.18) for boiled/baked/mashed potatoes, and 1.66 (95% CI: 1.43, 1.94) for French fries. In our cohort of patients with MI, we observed a ≈50% higher T2DM risk when comparing upper vs. lower tertiles of total or boiled potato intake. However, our analysis was hampered by the relatively low number of T2DM cases (*n* = 186), and in continuous analysis (RCS) the significant increase in T2DM risk at higher intakes was no longer observed. Therefore, our results should be interpreted with caution.

Because of the observational design of our study, we should be aware of confounding and potential biases. Extensive data collection was performed in the Alpha Omega Cohort, which included lifestyle, diet, CVD risk factors, and medication use. We adjusted for a large number of potential confounders in multivariable analysis. We performed a detailed assessment of dietary intakes, such as potatoes and their preparation methods, by means of 203-item validated FFQ. However, this method was not suitable for measuring salt intake. Because in the traditional Dutch diet, potatoes are often salted during cooking, the lack of adjustment for salt intake could have biased the associations for potatoes in an unfavorable direction. When assessing diet by means of an FFQ, systematic errors may occur, such as under-reporting of energy dense products or over-reporting of healthy foods. The low consumption of French fries in our study could be due to (deliberate) under-reporting because patients with MI in the Netherlands are well aware that this is an unhealthy food choice. We consider over-reporting of healthy foods less likely since results remained similar when stratifying for adherence to dietary guidelines (DHD15-index).

All patients in our study had a history of MI, and it is possible that they changed their diets because of health complaints or upon doctor's advice. We, therefore, performed a series of sensitivity analyses in which we consecutively excluded the first 2 years of follow-up, patients with a recent MI (<1 year before study enrolment), and patients with poor/moderate self-rated health. Results remained similar, making bias due to pre-existing disease or deteriorating health unlikely. We cannot rule out misclassification of patients due to the lack of sufficient repeated dietary assessments, which could have led to attenuated risk estimates for potato intake. However, our sensitivity analysis with a shortened follow-up duration showed similar results with long-term follow-up indicating no dilution of the associations.

The Alpha Omega Cohort consists of Dutch post-MI patients, predominantly males, and cardiometabolic risk is largely controlled by state-of-the-art drug treatment. Medication use and the underlying disease process could have affected the patients' sensitivity to lifestyle and dietary aspects, such as potato consumption. Results of the current study in patients with MI cannot merely be translated to healthy populations and may also be less applicable to women and people of non-Caucasian origin.

In conclusion, we observed no associations for total and boiled potato consumption with CVD mortality risk in Dutch post-MI patients but possible higher risks for all-cause mortality and T2DM. These findings warrant confirmation in other patients with IHD cohorts.

## Data Availability Statement

The raw data supporting the conclusions of this article will be made available by the authors, without undue reservation.

## Ethics Statement

The studies involving human participants were reviewed and approved by a Central Ethics Committee (Haga Hospital) and by an Ethics Committee in each participating hospital. The patients/participants provided their written informed consent to participate in this study.

## Author Contributions

EC conducted the research, performed data-analysis, interpreted the data, wrote the final manuscript, and had primary responsibility for final content. II and AS performed data-analysis, drafted the first version of the manuscript, and critically reviewed the manuscript. MB supervised the data analysis and critically reviewed the manuscript. JG conceived and designed the study, performed data acquisition, critically reviewed the manuscript, and had primary responsibility for final content. All the authors have read and approved the final manuscript.

## Funding

The Alpha Omega Cohort was funded by the Netherlands Heart Foundation (Grant 200T401) and the National Institutes of Health (NHLBI/ODS Grant R01 HL 076200). The research presented in this paper was supported by the Jaap Schouten Foundation (JSF_SU_10_2018) and a grant from Regio Deal Foodvalley (162135). The funding sources had no role in the study design and conduct, data analysis, or manuscript preparation.

## Conflict of Interest

The authors declare that the research was conducted in the absence of any commercial or financial relationships that could be construed as a potential conflict of interest.

## Publisher's Note

All claims expressed in this article are solely those of the authors and do not necessarily represent those of their affiliated organizations, or those of the publisher, the editors and the reviewers. Any product that may be evaluated in this article, or claim that may be made by its manufacturer, is not guaranteed or endorsed by the publisher.
