# Supplementary material for: Potato Consumption and Risk of Cardiovascular Mortality and Type 2 Diabetes After Myocardial Infarction: A Prospective Analysis in the Alpha Omega Cohort
Source: Front Nutr. 2022 Jan 27;8:813851. doi: 10.3389/fnut.2021.813851 (PMC8829223; doi:10.3389/fnut.2021.813851)
Supplement: Supplementary file 1 [file Data_Sheet_1.docx]

**Supplementary Material – Potato intake and cardiometabolic risk**

| **Supplemental Figures** | |  |
| --- | --- | --- |
| Supplemental Figure 1 | Flow diagram for selecting the population for analysis from the Alpha Omega Cohort. | 2 |
| Supplemental Figure 2 | Associations for total potatoes and T2DM incidence in 3,401 patients from the Alpha Omega Cohort from restricted cubic splines. | 3 |
| Supplemental Figure 3 | Hazard ratios per increment of total potato and CVD mortality in subgroups in 3,401 patients from the Alpha Omega Cohort. | 4 |
| Supplemental Figure 4 | Hazard ratios per increment of total potato and CVD mortality in subgroups in 3,401 patients from the Alpha Omega Cohort. | 5 |
| Supplemental Figure 5 | Hazard ratios per increment of total potato and all-cause mortality in subgroups in 3,401 patients from the Alpha Omega Cohort. | 6 |

**
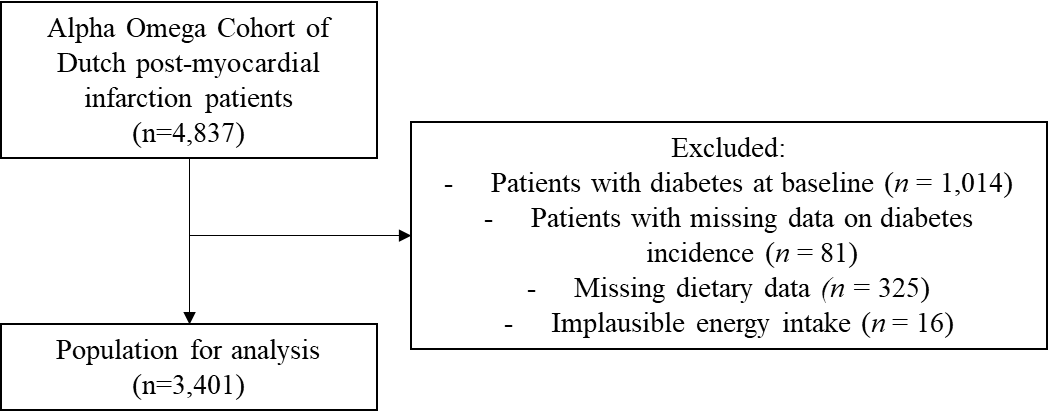
Supplemental Figure 1** Flow diagram for selecting the population for analysis from the Alpha Omega Cohort.


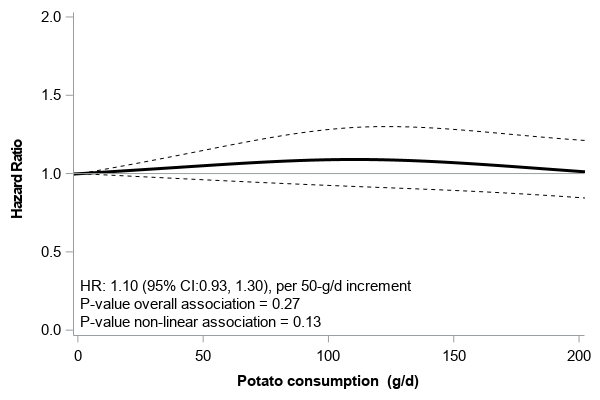


**Supplemental Figure 2** Associations for total potatoes and T2DM incidence in 3,401 patients from the Alpha Omega Cohort from restricted cubic splines.

Lines are restricted cubic splines, showing continuous associations, with 3 knots located at the 5^th^, 50^th^ and 95^th^ percentiles. The y-axis shows the predicted HRs for T2DM for any value of total potato intake, compared to the reference value set at 0 g/d. HRs are adjusted for age, sex, energy intake, education level, smoking, physical activity level, alcohol intake, whole and refined grains, fish, red and processed meat, milk, yogurt and custard, vegetables, fruits, mayonnaise, saturated fatty acids, poly unsaturated fatty acids, sugar sweetened beverages, sweet and sour snacks and nuts, seeds and legumes.


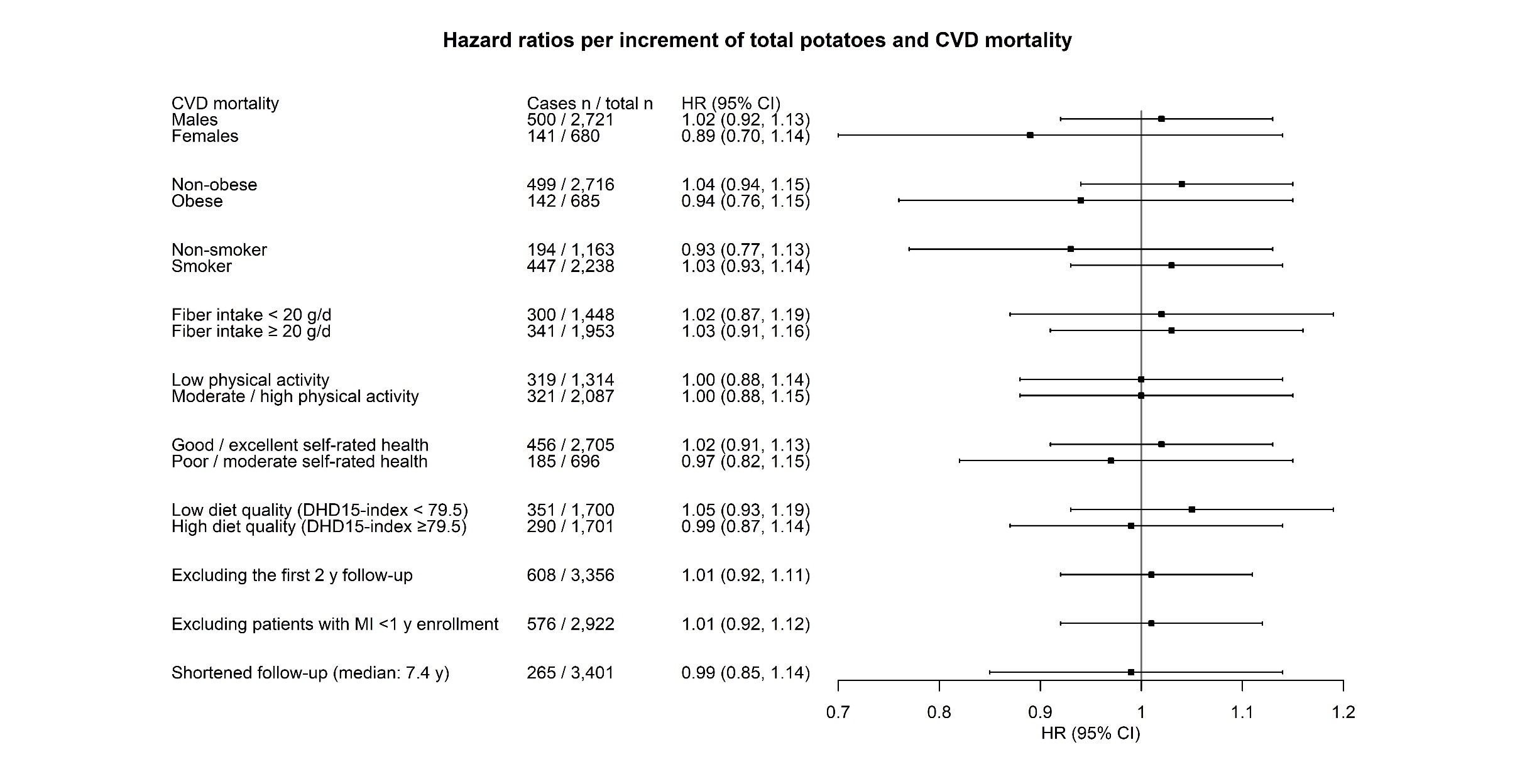
**Supplemental Figure 3** Hazard ratios per increment of total potato and cardiovascular disease mortality in subgroups in 3,401 patients from the Alpha Omega Cohort.

Hazard ratios (95% confidence intervals) obtained from Cox proportional hazards models, per 50 g/d increment of potato intake. HRs are adjusted for age, sex, energy intake, education level, smoking, physical activity level, alcohol intake, whole and refined grains, fish, red and processed meat, milk, yogurt and custard, vegetables, fruits, mayonnaise, saturated fatty acids, poly unsaturated fatty acids, sugar sweetened beverages, sweet and sour snacks and nuts, seeds and legumes.


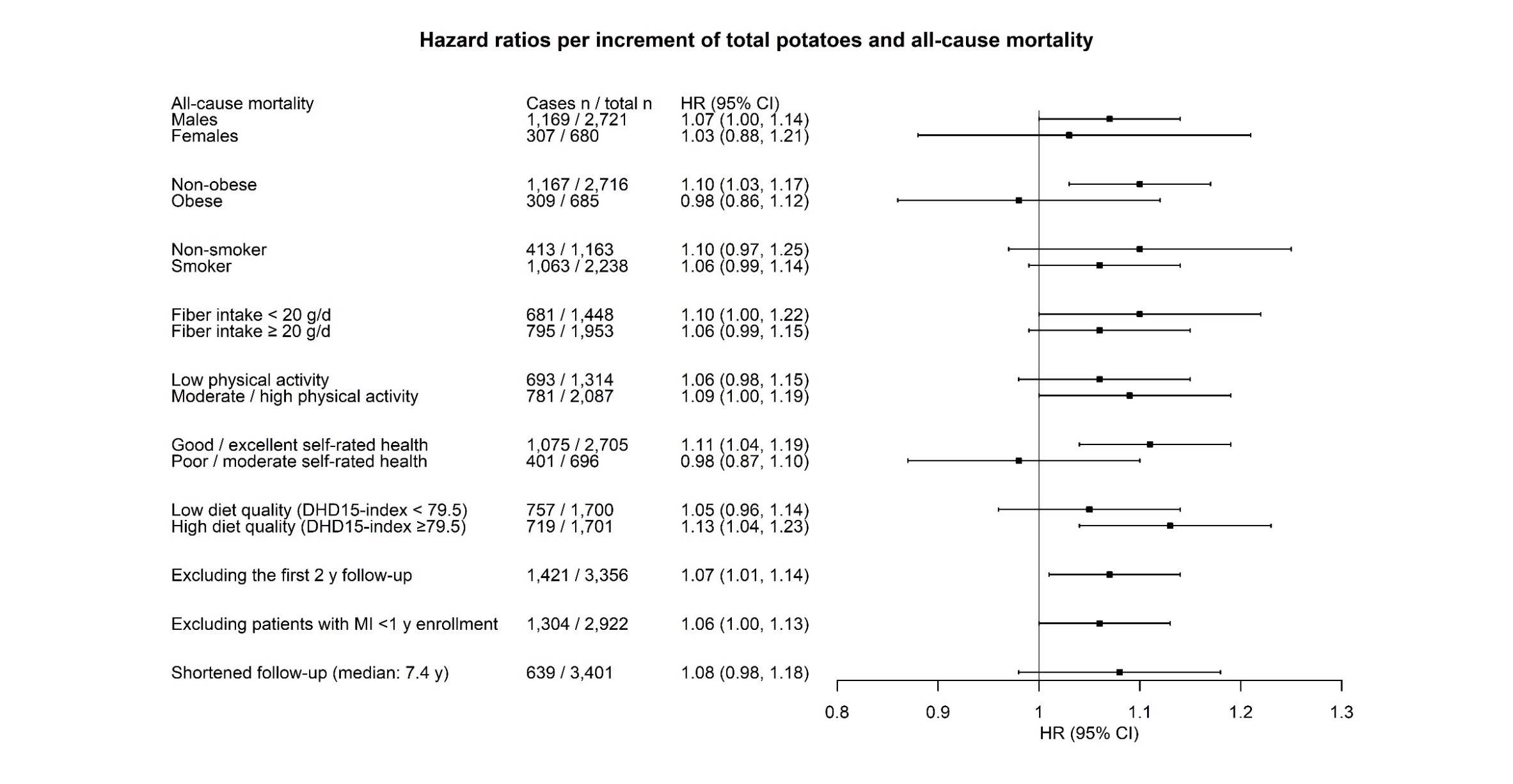


**Supplemental Figure 4** Hazard ratios per increment of total potato and all-cause mortality in subgroups in 3,401 patients from the Alpha Omega Cohort.

Hazard ratios (95% confidence intervals) obtained from Cox proportional hazards models, per 50 g/d increment of potato intake. HRs are adjusted for age, sex, energy intake, education level, smoking, physical activity level, alcohol intake, whole and refined grains, fish, red and processed meat, milk, yogurt and custard, vegetables, fruits, mayonnaise, saturated fatty acids, poly unsaturated fatty acids, sugar sweetened beverages, sweet and sour snacks and nuts, seeds and legumes.


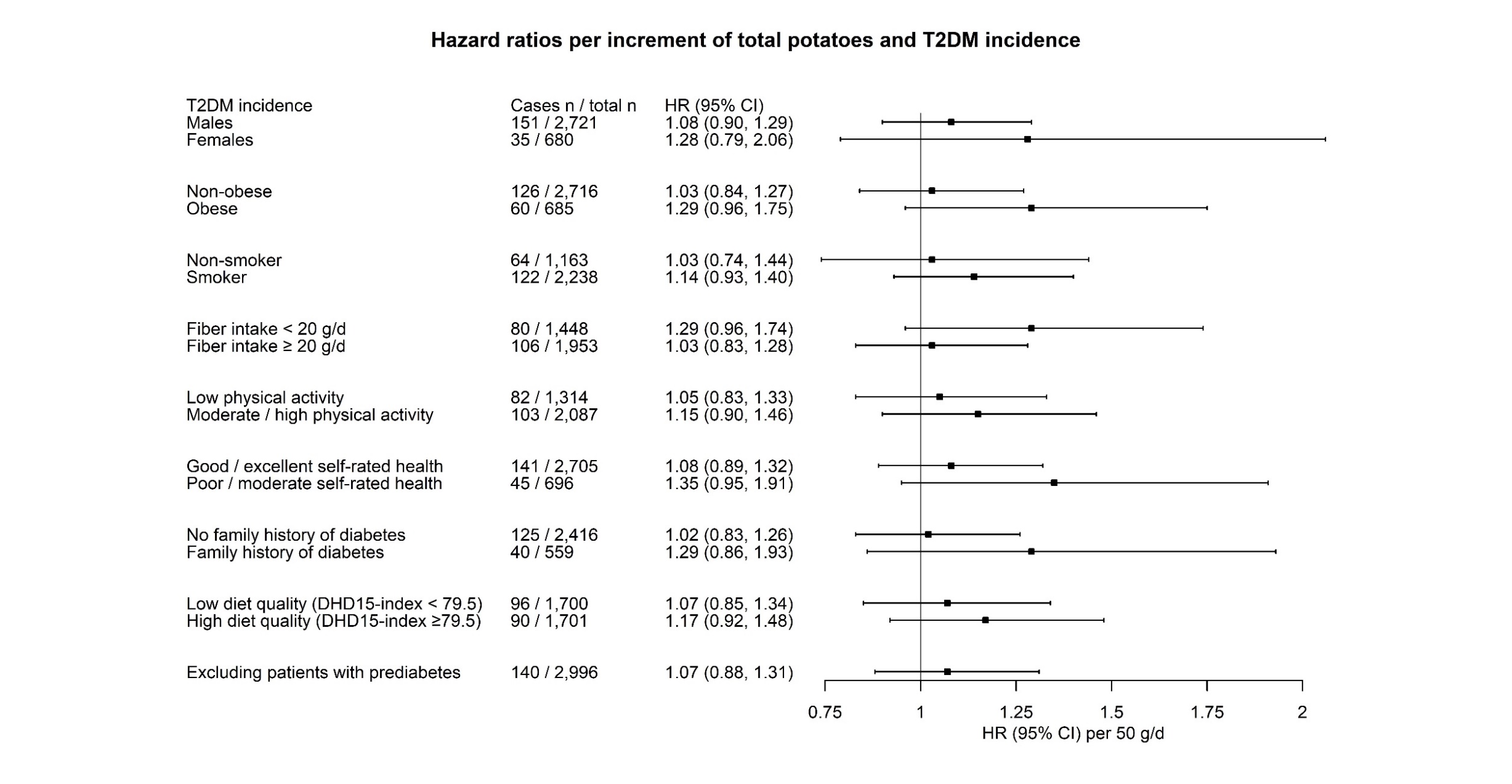
**Supplemental Figure 5** Hazard ratios per increment of total potato and T2DM incidence in subgroups in 3,401 patients from the Alpha Omega Cohort.

Hazard ratios (95% confidence intervals) obtained from Cox proportional hazards models, per 50 g/d increment of potato intake. HRs are adjusted for age, sex, energy intake, education level, smoking, physical activity level, alcohol intake, whole and refined grains, fish, red and processed meat, milk, yogurt and custard, vegetables, fruits, mayonnaise, saturated fatty acids, poly unsaturated fatty acids, sugar sweetened beverages, sweet and sour snacks and nuts, seeds and legumes.
